# Função do Ventrículo Direito e Estresse Oxidativo Melhoram com a Administração de Hormônios da Tireoide e Suco de Uva em um Modelo de Hipertensão Pulmonar

**DOI:** 10.36660/abc.20230602

**Published:** 2024-07-01

**Authors:** Isabel Proença, Patrick Turck, Vanessa Ortiz, Cristina Campos-Carraro, Adriane Bello Klein, Alexandre de Castro, Caroline Dani, Alex Sander da Rosa Araujo

**Affiliations:** 1 Universidade Federal do Rio Grande do Sul Porto Alegre RS Brasil Universidade Federal do Rio Grande do Sul, Porto Alegre, RS – Brasil

**Keywords:** Antioxidantes, Distúrbios do manejo do Cálcio, Monocrotalina, Fator 2 Relacionado a NF-E2

## Abstract

**Fundamento:**

A remodelação adversa dos vasos pulmonares eleva a pressão pulmonar e provoca hipertensão arterial pulmonar (HAP). A HAP resulta em aumento da pós-carga do ventrículo direito (VD), causando hipertrofia ventricular e consequente insuficiência cardíaca. Não existe um tratamento específico para o remodelamento desadaptativo do VD secundário à HAP.

**Objetivos:**

Este estudo tem como objetivo explorar duas abordagens terapêuticas, o suco de uva (SU) e os hormônios tireoidianos (HT), no tratamento do estresse oxidativo induzido pela HAP e nas alterações funcionais cardíacas.

**Métodos:**

Parâmetros ecocardiográficos relacionados à resistência dos vasos pulmonares (relação TA/TE), contratilidade do VD (ESPAT) e função diastólica do VD (relação dos picos E/A) foram avaliados. Além disso, foram medidos ROS totais, peroxidação lipídica, enzimas antioxidantes, proteínas de manipulação de cálcio, expressão de proteínas pró-oxidantes e antioxidantes. Valores de p<0,05 foram considerados estatisticamente significativos.

**Resultados:**

Ambos os tratamentos, com SU e HT, demonstraram uma redução na resistência pulmonar (~22%), além de melhorias na ESPAT (inotropismo ~11%) e na relação TA/TE (~26%) (p<0,05). Não houve alterações entre os grupos na relação do pico de E/A. Embora ROS e TBARS não tenham sido estatisticamente significativos, os tratamentos com SU e HT diminuíram os níveis de xantina oxidase (~49%) e normalizaram a expressão de HSP70 e proteínas de manipulação de cálcio (p<0,05). No entanto, apenas o tratamento com HT melhorou a função diastólica (~50%) e aumentou o imunoconteúdo de NRF2 (~48%) (p<0,05).

**Conclusões:**

Até onde sabemos, este estudo é pioneiro ao mostrar que o HT administrado em conjunto com o SU promoveu melhorias funcionais e bioquímicas em um modelo de HAP. Além disso, nossos dados sugerem que os tratamentos com SU e HT se mostraram cardioprotetores, sejam combinados ou não, e exibiram seus benefícios ao modular o estresse oxidativo e as proteínas de manipulação do cálcio.

## Graphical abstract

**Figura Central:**
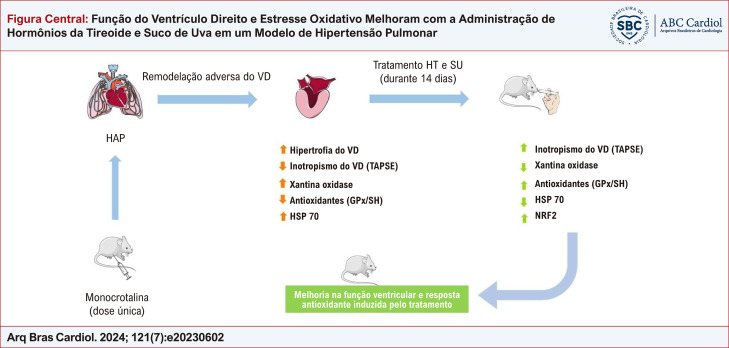
: Função do Ventrículo Direito e Estresse Oxidativo Melhoram com a Administração de Hormônios da Tireoide e Suco de Uva em um Modelo de Hipertensão Pulmonar

*O tratamento com hormônios tireoidianos (HT) e suco de uva (SU) melhora a contratilidade e a homeostase redox, conforme indicado pelo aumento da ESPAT (excursão sistólica do plano anular tricúspide), NRF2, glutationa peroxidase (GPx), níveis de sulfidrila (SH) total e redução de xantina oxidase (XO), usando ventrículo direito (VD), no modelo de hipertensão arterial pulmonar (HAP) induzida por monocrotalina. Partes da figura foram desenhadas usando imagens da Servier Medical Art. A Servier Medical Art da Servier é licenciada sob Creative Commons, com licença não portada de atribuição 3.0 (https://creativecommons.org/licenses/by/3.0/).*

## Introdução

A hipertensão pulmonar (HAP) é uma condição patológica que afeta aproximadamente 15-26 por milhão de adultos.^1,2^ A HAP leva à vasoconstrição pulmonar e ao aumento da resistência vascular pulmonar (RVP).^3^ A RVP elevada resulta em sobrecarga pressórica, causando dilatação, hipertrofia e disfunção do ventrículo direito (VD), conhecida como *cor pulmonale.*^4^ Essa disfunção resulta do comprometimento de proteínas manipuladoras de cálcio, como ATPase de cálcio do retículo sarcoplasmático (SERCA), receptor de rianodina e fosfolambam, devido ao aumento da pós-carga ventricular.^5,6^ O estresse oxidativo tem sido considerado um importante fator mecanicista envolvido na progressão da hipertrofia para a insuficiência do VD.

A perturbação da homeostase redox está associada ao remodelamento adverso do VD e à progressão para a insuficiência cardíaca.^7^ Além da produção mitocondrial de ROS, outras fontes apresentam papéis importantes na geração de radicais livres, como a xantina oxidase.^8^ Por outro lado, a resposta antioxidante é estimulada pelo fator de transcrição nuclear eritroide 2 (NFE2), relacionado ao fator 2 (NRF2), que reconhece o elemento responsivo antioxidante (ERA) comum do DNA e inicia a transcrição de genes antioxidantes. Condições de estresse, hormônios e compostos fenólicos podem induzir a expressão de NRF2 e aumentar a capacidade antioxidante celular.

Nesse contexto, foi descrito anteriormente que o consumo de suco de uva (SU) induz efeitos vasodilatadores, que a presença de diversas moléculas antioxidantes, como catequina, quercetina e antocianidina, pode explicar.^9,10^ Os polifenóis atuam como moléculas sinalizadoras, modulando a expressão de NRF2 e afetando o estado redox celular.^11^ Além desses compostos, os hormônios tireoidianos (HT) também podem influenciar a ativação do NRF2.^12^ Além disso, os HT têm sido reconhecidos como cardioprotetores em modelos de infarto cardíaco, uma vez que o tratamento com HT melhorou a contratilidade ventricular em ratos infartados.^13^

Os HT exercem efeito protetor sobre o ventrículo e o SU apresenta ação relevante como vasodilatador e, portanto, o objetivo do estudo foi avaliar os efeitos da administração de SU e HT, isolados e combinados, sobre a remodelação cardiovascular, bem como sobre o estresse oxidativo em modelo experimental de HAP.

## Métodos

### Considerações éticas e grupos experimentais

Ratos Wistar machos foram cedidos pelo Centro de Reprodução e Experimentação de Animais de Laboratório (CREAL) da Universidade Federal do Rio Grande do Sul (UFRGS). A pesquisa foi conduzida de acordo com o Comitê de Ética no Uso de Animais (CEUA – UFRGS) (número de aprovação: 37372). Os animais foram divididos em cinco grupos experimentais (N=46): o grupo controle recebeu injeção intraperitoneal de soro fisiológico e água por gavagem; o grupo HAP recebeu injeção de monocrotalina (MCT); o grupo HAP+SU recebeu MCT e suco de uva por gavagem; o grupo HAP+HT recebeu MCT e T3/T4 por gavagem; o grupo HAP+HT+SU recebeu MCT, hormônios tireoidianos e, uma hora após esta administração, suco de uva. No 21º dia experimental, um ecocardiograma foi realizado, seguido de eutanásia. De acordo com o método de randomização, os animais foram numerados de 1 a 46 e o sorteio dos números foi realizado manualmente para compor os grupos experimentais. As análises foram realizadas de forma duplo-cega.

### Determinação do tamanho da amostra

O tamanho da amostra foi estimado utilizando o software Sigma Plot 11.0. A probabilidade de erro α = 0,05 e o poder estatístico do teste (probabilidade de erro 1-β) = 0,95 foram considerados. O n calculado foi de 10 amostras por grupo experimental (considerando três protocolos experimentais distintos) utilizando a pressão sistólica intraventricular direita como desfecho principal.

### HAP induzida por MCT

No primeiro dia do protocolo experimental, uma dose única de MCT (60 mg/kg intraperitoneal) foi administrada a ratos Wistar (180-220 g) para induzir a HAP.

### Tratamento com suco orgânico de uva roxa

O SU integral orgânico foi obtido da empresa *Uva’só* Produtos Orgânicos (Garibaldi, RS, Brasil). O conteúdo total de compostos fenólicos analisados resultou em 4.052,2 mg/L (flavonoides (87%), antocianinas (13%) e resveratrol (0,01%)). Os animais receberam SU por gavagem na dose de 7 µL/g. A administração do suco foi iniciada sete dias após a injeção do MCT e durou 14 dias, até o final do protocolo experimental. A dose de SU foi baseada em estudos anteriores, que mostram que 7 µl/g equivale a cerca de 400 mL de suco (aproximadamente dois copos de 200 mL de suco/dia) para um adulto com peso de 70 kg.^14^

### Administração de HT

A administração de HT (T3 (2μg/100g/dia) e T4 (8μg/100g/dia) por gavagem) começou sete dias após a injeção do MCT e durou 14 dias, até o final do protocolo experimental.^13^

### Análise ecocardiográfica e morfométrica

Os animais foram anestesiados (cetamina 90 mg/kg; xilazina 10 mg/kg, intraperitoneal). As imagens foram obtidas por meio de Doppler bidimensional, modo M e pulsado (Philips HD7 Ultrasound System, Andover, MA, EUA), com transdutor S12-4 (Philips, Andover, MA, EUA). A relação entre o tempo de aceleração e o tempo de ejeção do fluxo sanguíneo pela artéria pulmonar (TA/TE), a excursão sistólica do plano anular tricúspide (ESPAT) e a taxa de fluxo durante o enchimento rápido e lento do VD (relação entre picos E/A) foram avaliadas. O índice de Fulton foi calculado usando peso do VD/peso (ventrículo esquerdo (VE) + septo (S)).

### Coleta da amostra

Após avaliação ecocardiográfica, os animais foram eutanasiados, o VD foi dissecado e armazenado a -80 °C.

### Avaliação do estresse oxidativo

A concentração de radicais livres totais foi determinada pelo método de fluorescência por meio da reação com diacetato de diclorofluoresceína (DCFH-DA)^15^ (nmol/mg de proteína). O dano lipídico foi avaliado pela determinação da concentração de substâncias reativas ao ácido tiobarbitúrico (TBARS) (nmol/mg de proteína).^16^

### Determinação da resposta antioxidante

A quantificação da atividade da superóxido dismutase (SOD) foi baseada na capacidade da SOD em inibir a auto-oxidação do pirogalol (unidades de SOD/mg de proteína).^17^ A atividade da catalase (CAT) foi medida pelo consumo de H_2_O_2_^18^ (pmol CAT/mg proteína). A glutationa peroxidase (GPx) foi determinada proporcionalmente ao consumo de NADPH (nmol por minuto/mg de proteína).^19^ O conteúdo total de sulfidrila foi medido pela reação com DTNB (nmol TNB/mg de proteína).^20^

### Análise de Western Blot

As amostras foram submetidas à eletroforese em gel de poliacrilamida (8-14%). As proteínas foram transferidas para uma membrana de difluoreto de polivinilideno (membrana de transferência Immobilon-P; Millipore). A imunodetecção foi realizada utilizando os seguintes anticorpos: xantina oxidase (XO) (150 kDa) (H-110): sc-20991, Lote: D1511; HSP 70 (70 kDa) (K-20): as-1060; Lote: G3013; SOD2 (25kDa) (G-20): as-18504, Lote: G1013; catalase (64kDa) (H-300): sc-50508, Lote: L2812; NRF2 (57 kDa) (c-20): sc-722; Lote: h1613; p-fosfolambam (25 kDa) (Trh 17): sc-17024-R, Lote: K2409; fosfolambam (25 kDa) (FL-52): sc-30142, Lote: H1908; SERCA (100 kDa) (H-300): sc-30110, Lote: L0205; e rianodina (42 kDa) (H-300): sc-13942, Lote: F1808. Todos os anticorpos foram adquiridos da Santa Cruz Biotechnology. Anticorpos secundários (conjugado de peroxidase de rabanete anti-cabra ou anti-coelho) foram utilizados para detecção por quimioluminescência no sistema Quant LAS4000 (GE Healthcare) e a expressão foi quantificada utilizando o software ImageJ. O método de Ponceau foi utilizado para normalização.^21^ O tamanho da amostra para cada proteína foi de quatro animais por grupo, escolhidos aleatoriamente.

### Análise estatística

A distribuição dos dados foi determinada pelo teste de Shapiro-Wilks. Para os dados com distribuição normal de variância homogênea, utilizamos a análise de variância (ANOVA) unidirecional, seguida do pós-teste de Tukey (F). Para os dados com distribuição normal e variância não homogênea, utilizamos a análise de variância de Welch (ANOVA), seguida do pós-teste de Games Holmes (W), os quais foram apresentados como média ± desvio padrão. Para os dados que não apresentaram distribuição normal, utilizamos a análise de Kruskal-Wallis com pós-teste de Dunn (K), e os dados foram apresentados como mediana e percentis 25 e 75. Valores de p<0,05 foram considerados estatisticamente significativos. Todas as análises foram realizadas no software SPSS Statistic 18. O teste de Grubbs do software GraphPad Prism foi utilizado para determinar valores discrepantes, que foram retirados das análises estatísticas.

## Resultados

### Avaliação funcional e morfométrica do VD

**Figura 1:**
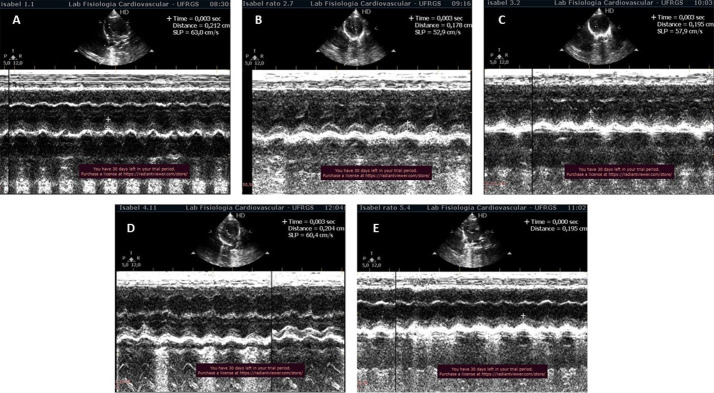
– Imagem representativa dos dados ecocardiográficos do VD. O ESPAT está marcado para os grupos (A) CTR: Controle, (B) HAP: hipertensão arterial pulmonar; (C) HAP+SU: Hipertensão Arterial Pulmonar mais Suco de Uva, (D) HAP+HT: Hipertensão Arterial Pulmonar mais Hormônios Tireoidianos, e (E) HAP+HT+SU: Hipertensão Arterial Pulmonar mais Hormônios Tireoidianos mais Suco de Uva.

Considerando os parâmetros ecocardiográficos, observamos que a condição de HAP reduziu a relação TA/TE, conforme demonstrado pelos grupos HAP e HAP+HT+SU. No entanto, os tratamentos isolados foram eficazes na melhoria deste parâmetro, como observado nos grupos HAP+SU e HAP+HT (p<0,05). Da mesma forma, os tratamentos atenuaram a diminuição da ESPAT, que foi observada apenas no grupo HAP (p<0,05). Sobre a relação dos picos E/A do VD, houve diminuição no grupo HAP; apenas o grupo HAP+HT apresentou melhora em relação ao grupo HAP (p<0,05) (Tabela 1 e Figura 1).

**Tabela 1:** – Dados morfométricos e ecocardiográficos

Parâmetros	Controle (n=10)	HAP (n=10)	HAP+SU (n=9)	HAP+HT (n=7)	HAP+HT+SU (n=10)	Valor p
**Dados morfométricos**						
Peso VD (g)	0,64 [0,61-0,65]	0,56 [0,53-0,60]	0,53 [0,52-0,65]	0,62 [0,59-0,66]	0,60 [0,58-0,64]	0,048
Índice de Fulton (VD/VE+S)	0,27 ± 0,02	0,42 ± 0,07*	0,40 ± 0,07*	0,48 ± 0,10*	0,42 ± 0,07*	≤0,001
**Dados Ecocardiográficos**						
TA/TE	0,26 ± 0,04	0,15 ± 0,05*	0,19 ± 0,07	0,20 ± 0,08	0,15 ± 0,03*	0,002
ESPAT (cm)	0,21 [0,21-0,22]	0,18 [0,16-0,19]*	0,19 [0,18-0,21]	0,20 [0,18-0,21]	0,19 [0,18-0,19]	0,002
E/A	1,12 ± 0,12	0,76 ± 0,06*	0,79 ± 0,15*	1,27 ± 0,46	0,73 ± 0,09*	≤0,000

HAP: hipertensão arterial pulmonar; SU: suco de uva; HT: hormônios tireoidianos; VD: ventrículo direito; VE: ventrículo esquerdo; TA/TE: razão entre o tempo de aceleração e o tempo de ejeção do fluxo sanguíneo pela artéria pulmonar; ESPAT: excursão sistólica do plano do anel tricúspide; E/A: velocidade de enchimento ventricular precoce/velocidade de enchimento ventricular tardio para o VD. ANOVA unidirecional (pós-teste de Tukey), dados apresentados como média ± desvio padrão. Teste de Kruskal-Wallis (pós-Dunn), dados apresentados como mediana e percentis 25 e 75. *diferença significativa em relação ao grupo controle.

Ao analisar o índice de Fulton, o grupo HAP+HT apresentou aumento significativo em relação ao grupo HAP+SU (p<0,05). Não foi observada diferença significativa no pós-teste em relação ao peso do VE. Porém, foi observado aumento do índice de Fulton em todos os grupos submetidos à HAP em relação ao controle (p<0,05) (Tabela 1).

### Marcadores de estresse oxidativo

**Figura 2:**
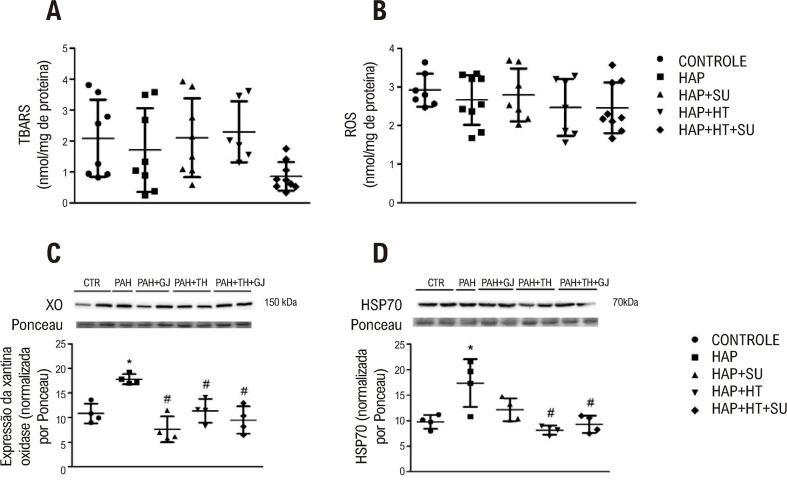
– Análise de marcadores de estresse oxidativo (A) TBARS, (B) ROS, (C) XO, e (D) HSP70. Os dados foram expressos em média e desvio padrão (n=9-4/grupo). (*) Diferença significativa em relação ao grupo controle. (#) Diferença significativa em relação ao grupo HAP. Análise estatística de (A) pela ANOVA de Welch com pós-teste de Games Holmes e de (B), (C), (D) e (E) ANOVA unidirecional com pós-teste de Tukey (p<0,05). CTR: Controle; HAP: hipertensão arterial pulmonar; HAP+SU: Hipertensão Arterial Pulmonar mais Suco de Uva; HAP+HT: hipertensão arterial pulmonar mais hormônios tireoidianos; HAP+HT+SU: hipertensão arterial pulmonar mais hormônios tireoidianos mais suco de uva.

Em relação aos níveis de TBARS e ROS, não houve diferença significativa entre os grupos (Figura 2A e B). A enzima pró-oxidante xantina oxidase (XO) apresentou expressão proteica aumentada no grupo HAP em relação ao controle. No entanto, foi detectado um efeito positivo dos tratamentos, pois os grupos tratados apresentaram uma diminuição nos níveis de XO nos grupos HAP+SU, HAP+HT e HAP+HT+SU em relação ao grupo HAP (p<0,05) (Figura 2C). Além disso, o grupo HAP apresentou expressão elevada de HSP70 em comparação com o grupo controle. Porém, os grupos HAP+HT e HAP+HT+SU apresentaram diminuição na expressão de HSP70 em relação ao grupo HAP (p<0,05) (Figura 2D).

### Resposta antioxidante

**Figura 3:**
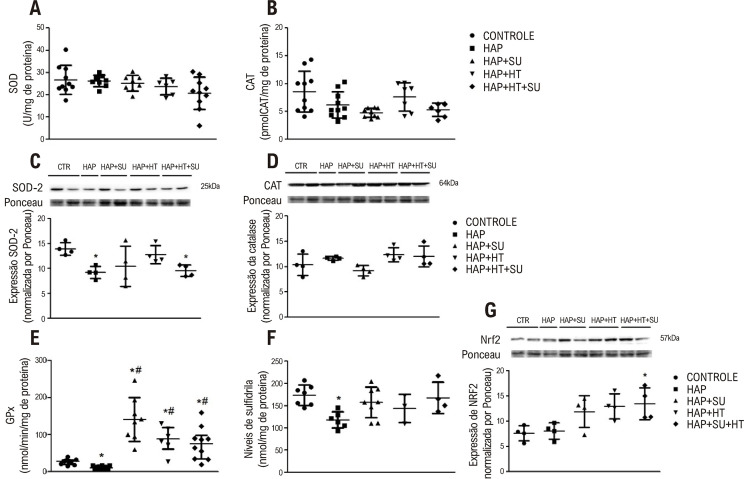
– Resposta antioxidante: (A) atividade SOD, (B) atividade CAT, (C) expressão da proteína SOD2, (D) expressão da proteína CAT, (E) atividade GPX, (F) grupo Sulfidril e (G) expressão da proteína NRF2. Os dados foram expressos em média e desvio padrão (n=10-4/grupo). (*) Diferença significativa em relação ao grupo controle. (#) Diferença significativa em relação ao grupo HAP. Análise estatística de (B), (C) e (E) pela ANOVA de Welch com pós-teste de Games Holmes e de (A), (C), (D), (F) e (G) ANOVA unidirecional com Pós-teste de Tukey (p< 0,05). CTR: controle; HAP: hipertensão arterial pulmonar; HAP+SU: hipertensão arterial pulmonar mais suco de uva; HAP+HT: hipertensão arterial pulmonar mais hormônios tireoidianos; HAP+HT+SU: hipertensão arterial pulmonar mais hormônios tireoidianos mais suco de uva.

Ao avaliarmos os antioxidantes enzimáticos, a atividade de SOD e CAT não apresentou diferenças entre os grupos (Figura 3A e B). A expressão da proteína SOD2 foi diminuída nos grupos HAP e HAP+HT+SU em relação ao controle (p<0,05). A expressão da catalase não foi diferente entre os grupos (Figura 3C e D). Considerando a atividade da glutationa peroxidase (GPx), os animais com HAP apresentaram diminuição quando comparados ao controle. Porém, a atividade dessa enzima foi aumentada nos grupos HAP+SU, HAP+HT e HAP+HT+SU em relação aos grupos controle e HAP (p<0,05). Além disso, houve diminuição nos níveis de grupos sulfidrila nos grupos HAP em relação ao controle, HAP+SU, HAP+HT e HAP+HT+SU (p<0,05) (Figura 3E e F). Como o NRF2 é capaz de controlar a transcrição de diversas enzimas antioxidantes, a expressão desse fator foi avaliada. O grupo HAP+HT+SU apresentou níveis aumentados de proteína NRF2 em comparação com o grupo controle (p<0,05) (Figura 3G).

### Proteínas de manejo de cálcio

**Figura 4:**
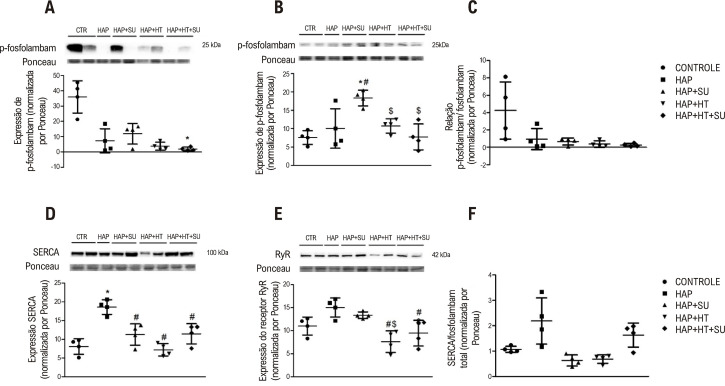
– Proteína de manejo do cálcio. (A) P-fosfolambam, (B) fosfolambam total, (C) Razão P-fosfolambam/Fosfolambam total, (D) SERCA, (E) Receptor de rianodina e (F) Razão SERCA/fosfolambam total. Os dados foram expressos em média e desvio padrão (n=4/grupo). (*) Diferença significativa em relação ao grupo controle. (#) Diferença significativa em relação ao grupo HAP. ($) Diferença significativa em relação ao grupo HAP+SU. Análise estatística de (A) por Kruskal-Wallis com pós-teste de Dunn, de (B), (D) e (E) por ANOVA unidirecional com pós-teste de Tukey e de (C) e (F) por ANOVA de Welch com pós-teste de Games Holmes (p<0,05). CTR: controle; HAP: hipertensão arterial pulmonar; HAP+SU: hipertensão arterial pulmonar mais suco de uva; HAP+HT: hipertensão arterial pulmonar mais hormônios tireoidianos; HAP+HT+SU: hipertensão arterial pulmonar mais hormônios tireoidianos mais suco de uva.

Em relação à expressão da proteína p-fosfolambam, foi observada diminuição no grupo co-tratado em comparação ao grupo controle (p<0,05) (Figura 4A). Quanto aos níveis de fosfolambam total, houve aumento no grupo HAP+SU em relação aos grupos controle e HAP. Por outro lado, esta proteína foi diminuída nos grupos HAP+HT e HAP+HT+SU em comparação ao grupo HAP+SU (p<0,05) (Figura 4B). Apesar disso, quando avaliada a relação p-fosfolambam/fosfolambam, não houve alteração significativa entre os grupos (Figura 4C). Em relação à expressão da SERCA, houve aumento nos níveis proteicos no grupo HAP; entretanto, os grupos HAP+SU, HAP+HT e HAP+HT+SU apresentaram expressão diminuída da proteína em relação ao grupo HAP (p<0,05). Em relação à expressão da proteína receptora de rianodina, houve diminuição nos grupos HAP+HT e HAP+HT+SU em relação aos grupos HAP e HAP+SU (p<0,05) (Figura 4D e E). Sobre a relação SERCA/fosfolambam total, não houve diferença entre os grupos (Figura 4F).

## Discussão

O presente trabalho é inovador ao considerarmos que, pela primeira vez, descreveu a associação entre o efeito protetor do SU nos vasos pulmonares e a ação do HT contra o remodelamento adverso do VD em um modelo de HAP. Em nosso protocolo experimental, tanto SU quanto HT melhoraram parâmetros funcionais, como as relações ESPAT, TA/TE e E/A, além de modularem a expressão de proteínas reguladoras de cálcio. Em relação ao estresse oxidativo, a xantina oxidase (XO), a proteína de choque térmico 70 kDa (HSP70) e a NrF2 foram responsivas à administração de SU e HT, o que culminou na restauração da homeostase redox (Figura Central).

A HAP induziu a hipertrofia do VD, o que corrobora outros estudos utilizando o mesmo protocolo experimental.^22^ Embora a resposta hipertrófica do VD seja uma tentativa de superar o aumento da pós-carga cardíaca causado pela hipertensão pulmonar; suas consequências a longo prazo são deletérias à função cardíaca.^23^ Essa hipertrofia está associada a alterações na relação TA/TE e E/A, bem como no ESPAT. O LA reduzido representa um fechamento prematuro da valva pulmonar devido à hipertensão. Ratos com HAP também exibem uma extensão da TE, levando a uma relação TA/TE reduzida neste grupo. A análise ESPAT, por outro lado, representa uma forma de avaliar a contratilidade do VD.^24^ A HAP apresentou redução no ESPAT, indicando que o VD diminuiu a função contrátil. Além disso, a relação E/A apresentou redução significativa no grupo HAP, indicando diminuição da complacência ventricular e comprometimento da função diastólica. Tomados em conjunto, estes dados ecocardiográficos corroboram o fato que a HAP leva à insuficiência do VD. O tratamento da HAP com SU e HT demonstrou melhora nos parâmetros funcionais, já que, nos grupos HAP+HT e HAP+SU, a relação TA/TE não foi diferente do controle, significando menor resistência na vasculatura pulmonar. Ludke et al*.*^25^ também demonstraram que o tratamento por seis semanas com SU evitou as alterações na relação TA/TE induzidas pela HAP.^25,26^ Em relação ao ESPAT, ambos os tratamentos, isolados ou combinados, melhoraram a contratilidade do VD. A melhora do inotropismo pode estar associada a alterações na expressão das proteínas de manejo do cálcio. Nossos resultados mostraram uma normalização da expressão das proteínas de manejo do cálcio, principalmente no grupo HAP+SU+HT, apresentando valores mais próximos aos encontrados no grupo controle. Isso sugere que a combinação de tratamentos parece evidenciar um potencial cardioprotetor na HAP. As proteínas de manipulação de cálcio também foram sensíveis ao redox, indicando o papel crítico das ROS no modelo de HAP.

Mosele et al.^27^ e Castro et al.^13^ mostraram que a proteção do sistema cardiovascular pelo tratamento com SU e HT, respectivamente, envolveu a atenuação do estresse oxidativo. Em nosso estudo, os níveis de sulfidrila diminuíram em ratos com HAP, indicando redução na capacidade antioxidante não enzimática desses animais. Este contexto pode ser prejudicial, uma vez que os HAP diminuíram a expressão de SOD, reduzindo a proteção antioxidante não enzimática e enzimática. Por outro lado, embora os níveis reduzidos de SOD também tenham diminuído no HAP+ HT+SU, o HT mais o SU preveniram a diminuição dos níveis de sulfidrila e contra-regularam a resposta antioxidante ao estresse oxidativo imposto pela HAP.

Os radicais ânion superóxido são convertidos em peróxido de hidrogênio, que pode desempenhar um papel importante em muitas vias de sinalização.^28^ No entanto, os níveis suprafisiológicos de peróxido de hidrogênio podem superar a resposta antioxidante e perturbar a homeostase redox.^29^ CAT e GPx são relevantes para manter níveis adequados desta ROS. A HAP reduziu a atividade da GPx, implicando uma resposta antioxidante inadequada. Um perfil semelhante foi encontrado por Dos Santos Lacerda et al.,^30^ em cujo estudo a HAP levou à redução dos níveis de GPx. Além disso, Sun et al.^31^ mostraram que pacientes com HAP associada à esclerose sistêmica apresentaram GPx reduzido. SU e HT, isolados ou combinados, foram eficazes no aumento da GPx em nosso estudo. Corroborando esse resultado, em modelo experimental gestacional, Proença et al. demonstraram que o tratamento com SU protegeu o coração do feto contra o estresse oxidativo por meio de melhorias nos níveis de GPx.^32^ Bedê et al.^33^também observaram melhorias nos antioxidantes e aumento dos níveis de GPx após administração de SU em ratos tratados com dieta rica em gordura. Esses dados estão associados ao aumento dos níveis de NrF2 induzido pelo tratamento com HT e SU em nosso estudo, uma vez que a ativação de NrF2 resulta em maior expressão de enzimas antioxidantes e desencadeia efeitos citoprotetores.^34^ Assim, a modulação do NrF2 pode ser sugerida como mecanismo de ação do SU e HT na cardioproteção.

O presente estudo também avaliou a expressão de XO, proteína relacionada à produção de ROS, que estava aumentada no grupo HAP. Como fonte de ROS, um trabalho anterior descreveu uma interação molecular entre XO e TLR4 na ativação de neutrófilos, induzindo a translocação de NF-kB e promovendo inflamação.^35^ Ambos os tratamentos SU e HT, isolados ou combinados, foram eficazes na redução da expressão de XO. Corroborando isso, Castro et al.^13^ observaram redução na expressão de XO em ratos infartados tratados com HT; esse resultado foi correlacionado com a diminuição da expressão de MyD88, proteína relacionada ao processo inflamatório.^36^

A ruptura na homeostase redox desencadeia uma resposta ao estresse que envolve o recrutamento de proteínas chaperonas.^37^ Intracelularmente, a HSP70 desempenha papel relevante como chaperona, promovendo o enovelamento e redobramento de proteínas, além de exibir efeito anti-inflamatório e atenuar o estresse celular. Em nosso estudo, observamos um elevado imunoconteúdo de HSP70 no grupo HAP. O aumento na expressão desta proteína pode indicar um mecanismo compensatório ao dano cardíaco induzido pela HAP. A administração de SU e HT reduziu os níveis de HSP70, indicando um ambiente celular menos oxidado.

### Limitação do estudo

A avaliação histológica do VD, para verificar o tamanho dos cardiomiócitos e a deposição de colágeno, seria importante no estudo do remodelamento cardíaco induzido pela HAP. Nesse sentido, consideramos a ausência desta avaliação no protocolo experimental uma limitação do estudo. Além disso, este relatório continua em fase de protocolo experimental e necessita de mais estudos para compreender os mecanismos relacionados à eficácia e segurança desta abordagem terapêutica.

## Conclusão

Nosso estudo demonstra, pela primeira vez, que o tratamento com SU com HT, isolado ou combinado, pode melhorar parâmetros vasculares funcionais do VD e pulmonares de ratos com HAP. Essa proteção cardiovascular pode estar associada à modulação da expressão das proteínas de manejo do cálcio, melhorando o inotropismo do VD e promovendo o restabelecimento da homeostase redox celular. Estes resultados são relevantes e abrem uma perspectiva de novas estratégias terapêuticas para mitigar as complicações cardiovasculares da HAP.
