# Supplementary material for: Função do Ventrículo Direito e Estresse Oxidativo Melhoram com a Administração de Hormônios da Tireoide e Suco de Uva em um Modelo de Hipertensão Pulmonar
Source: Arq Bras Cardiol. 2024 Jul 1;121(7):e20230602. [Article in Portuguese] doi: 10.36660/abc.20230602 (PMC12080604; doi:10.36660/abc.20230602)
Supplement: Translation

# Translation: Right Ventricular Function and Oxidative Stress Improve with the Administration of Thyroid Hormones and Grape Juice in a Pulmonary Hypertension Model

**DOI:** 10.36660/abc.20230602i

**Authors:** Isabel Proença, Patrick Turck, Vanessa Ortiz, Cristina Campos-Carraro, Adriane Bello Klein, Alexandre de Castro, Caroline Dani, Alex Sander da Rosa Araujo

**Affiliations:** 1 Brazil Universidade Federal do Rio Grande do Sul, Porto Alegre, RS – Brazil

**Keywords:** Antioxidants, Calcium Handling Disorders, Monocrotaline, NF-E2-Related Factor 2

## Abstract

**Background:**

Adverse remodeling of lung vessels elevates pulmonary pressure and provokes pulmonary arterial hypertension (PAH). PAH results in increased right ventricle (RV) afterload, causing ventricular hypertrophy and the onset of heart failure. There is no specific treatment for maladaptive RV remodeling secondary to PAH.

**Objectives:**

This study aims to explore two therapeutic approaches, grape juice (GJ) and thyroid hormones (TH), on PAH-induced oxidative stress and cardiac functional changes.

**Methods:**

Parameters of echocardiography related to lung vessel resistance (AT/ET ratio), RV contractility (TAPSE), and RV diastolic function (E/A peaks ratio) were evaluated. Also, total ROS, lipid peroxidation, antioxidant enzymes, calcium handling proteins, pro-oxidant and antioxidant protein expression were measured. Values of p<0.05 were considered statistically significant.

**Results:**

Both GJ and TH treatments demonstrated reductions in pulmonary resistance (~22%) and improvements in TAPSE (inotropism ~11%) and AT/ET ratio (~26%) (p<0.05). There were no changes amongst groups regarding the E/A peak ratio. Although ROS and TBARS were not statistically significant, GJ and TH treatments decreased xanthine oxidase (~49%) levels and normalized HSP70 and calcium handling protein expression (p<0.05). However, only TH treatment ameliorated diastolic function (~50%) and augmented NRF2 immunocontent (~48%) (p<0.05).

**Conclusions:**

To the best of our knowledge, this study stands as a pioneer in showing that TH administered together with GJ promoted functional and biochemical improvements in a PAH model. Moreover, our data suggest that GJ and TH treatments were cardioprotective, combined or not, and exhibited their beneficial effects by modulating oxidative stress and calcium-handling proteins.

## Introduction

Pulmonary hypertension (PAH) is a pathological condition that affects approximately 15-26 per million adults.^1,2^PAH exhibits pulmonary vasoconstriction, leading to increased pulmonary vascular resistance (PVR).^3^ The elevated PVR results in pressure overload, which causes dilatation, hypertrophy and dysfunction of the right ventricle (RV), known as c*or pulmonale.*^4^ This dysfunction results from the impairment of calcium-handling proteins such as sarcoplasmic reticulum calcium ATPase (SERCA), ryanodine receptor and phospholamban due to the increased ventricular afterload.^5,6^ Oxidative stress has been considered an important mechanical factor involved in the progression from hypertrophy to RV failure.

The disruption of redox homeostasis is related to the adverse remodeling of RV and progression to heart failure.^7^ In addition to the mitochondrial production of ROS, other sources exhibit important roles in free radical generation, such as xanthine oxidase.^8^ On the other hand, the antioxidant response is stimulated by the transcription factor nuclear erythroid 2 (NFE2)-related factor 2 (NRF2), which recognizes the common DNA antioxidant responsive element (ARE) and initiates the transcription of antioxidant genes. Stress conditions, hormones and phenolic compounds can induce NRF2 expression and increase cellular antioxidant capacity.

In this context, it was previously described that grape juice (GJ) consumption induces vasodilator effects, which the presence of many antioxidant molecules, such as catechin, quercetin, and anthocyanidin, in the GJ can explain.^9,10^ Polyphenols act as signaling molecules, modulating NRF2 expression and affecting cellular redox status.^11^ Besides these compounds, thyroid hormones (TH) can also influence NRF2 activation.^12^ Moreover, TH were recognized as cardioprotective in a cardiac infarction model, since treatment with TH improved ventricular contractility in infarcted rats.^13^

Since the TH exert a protective effect on the ventricle and the GJ shows a relevant action as a vasodilator, the objective of the study was to assess the effects of GJ and TH administration, isolated and combined, on cardiovascular remodeling and oxidative stress in an experimental model of PAH.

## Methods

### Ethical considerations and experimental groups

The Center for Reproduction and Experimentation of Laboratory Animals (CREAL) of the Federal University of Rio Grande do Sul (UFRGS) provided the male Wistar rats. The research was conducted in accordance with the Committee for Ethical Use of Animals (CEUA – UFRGS) (approval number: 37372). The animals were divided into five experimental groups (N=46): control received an intraperitoneal injection of saline and water by gavage; PAH received monocrotaline injection (MCT); PAH+GJ received MCT and grape juice by gavage; PAH+TH received MCT and T3/T4 by gavage; PAH+TH+GJ received MCT, thyroid hormones and, one hour after this administration, grape juice. On the 21^st^ experimental day, echocardiography was performed, followed by euthanasia. According to the randomization method, the animals were numbered from 1 to 46, and the number draw was performed manually to compose the experimental groups. The analyses were performed in a double-blind manner.

### Determination of sample size

The sample size was estimated using the Sigma Plot 11.0 Software. The probability of error α = 0.05 and statistical test power (1-β error probability) = 0.95 were considered. The calculated n was 10 samples per experimental group (considering three distinctive experimental protocols) using right intraventricular systolic pressure as the main outcome.

### MCT-induced PAH

On the first day of the experimental protocol, a single dose of MCT (60 mg/kg intraperitoneal) was administered to Wistar rats (180-220 g) to induce PAH.

### Organic red grape juice treatment

Organic whole GJ was obtained from *Uva’só* Organic Products (Garibaldi, RS, Brazil) company. The total phenolic compound content analyzed resulted in 4,052.2 mg/L (flavonoids (87%), anthocyanins (13%), and resveratrol (0.01%)). Animals received GJ via gavage at a dose of 7 µL/g. Juice administration started seven days after the MCT injection and lasted for 14 days until the end of the experimental protocol. The dose of GJ was based on previous studies showing that 7 µl/g accounts for circa 400 mL of juice (approximately two 200 mL glasses of juice/day) for an adult weighing 70 kg.^14^

### TH administration

TH administration (T3 (2μg/100g/day) and T4 (8μg/100g/day) by gavage) started seven days after MCT injection and lasted for 14 days until the end of the experimental protocol.^13^

### Echocardiographic and morphometric analysis

The animals were anesthetized (ketamine 90 mg/kg; xylazine 10 mg/kg, intraperitoneal). Images were obtained using two-dimensional, M-mode and pulsed Doppler (Philips HD7 Ultrasound System, Andover, MA, USA), with an S12-4 transducer (Philips, Andover, MA, USA). The ratio between the acceleration time and ejection time of blood flow through the pulmonary artery (AT/ET), the tricuspid annular plane systolic excursion (TAPSE) and the flow rate during fast and slow filling of the RV (E/A peaks ratio) were assessed. Fulton index was calculated using RV weight/(left ventricle (LV) + septum (S)) weight.

### Sample Collection

After echocardiography evaluation, animals were euthanized, the RV dissected, and stored at -80°C.

### Oxidative stress evaluation

The concentration of total free radicals was determined by the fluorescence method through reaction with dichlorofluorescein diacetate (DCFH-DA)^15^ (nmol/mg protein). Lipid damage was evaluated through determination of the concentration of thiobarbituric acid reactive substances (TBARS) (nmol/mg protein).^16^

### Determination of antioxidant response

The superoxide dismutase (SOD) activity quantification was based on the ability of SOD to inhibit pyrogallol auto-oxidation (units of SOD/mg protein).^17^ The catalase activity (CAT) was measured through the consumption of H_2_O_2_^18^ (pmol CAT/mg protein). Glutathione peroxidase (GPx) was determined proportionally to NADPH consumption (nmol per minute/mg protein).^19^ The total sulfhydryl content was measured by the reaction with DTNB (nmol TNB/mg protein).^20^

### Western Blot analysis

Samples were subjected to electrophoresis in polyacrylamide gel (8-14%). Proteins were transferred to a polyvinylidene difluoride membrane (Immobilon-P transfer membrane; Millipore). Immunodetection was performed using the following antibodies: xanthine oxidase (XO) (150 kDa) (H-110): sc-20991, Lot: D1511; HSP 70 (70 kDa) (K-20): as-1060, Lot: G3013; SOD2 (25kDa) (G-20): as-18504, Lot: G1013; catalase (64kDa) (H-300): sc-50508, Lot: L2812; NRF2 (57 kDa) (c-20): sc-722, Lot: h1613; p-phospholamban (25 kDa) (Trh 17): sc-17024-R, Lot: K2409; phospholamban (25 kDa) (FL-52): sc-30142, Lot: H1908; SERCA (100 kDa) (H-300): sc-30110, Lot: L0205; and ryanodine (42 kDa) (H-300): sc-13942, Lot: F1808. All antibodies were purchased from Santa Cruz Biotechnology. Secondary antibodies (anti-mouse, anti-goat or anti-rabbit radish peroxidase conjugate) were used for detection by chemiluminescence on Quant LAS4000 system (GE Healthcare), and expression was quantified using ImageJ software. The Ponceau method was used for normalization.^21^ The sample size for each protein was four animals per group, randomly chosen.

### Statistical analysis

Data distribution was determined using the Shapiro-Wilks test. For data showing normal distribution of homogeneous variance, one-way analysis of variance (ANOVA) was used, followed by Tukey’s post-test (F). For data showing normal distribution and non-homogeneous variance, Welch’s analysis of variance (ANOVA) was used, followed by the Games Holmes post-test (W), and presented as mean ± standard deviation. For data that did not show normal distribution, Kruskal-Wallis analysis with Dunn’s post-test was used (K), and the data was presented as median and 25^th^ and 75^th^ percentiles. Values of p<0.05 were considered statistically significant. All analyses were performed using SPSS Statistic 18 Software. The GraphPad Prism software’s Grubbs test was used to determine outlier values, which were removed from the statistical analyses.

## Results

### Functional and morphometric RV evaluation

Considering the echocardiographic parameters, we observed that the PAH condition reduced the AT/ET ratio, as shown by the PAH and PAH+TH+GJ groups. However, isolated treatments were effective in improving this parameter, as seen in the PAH+GJ and PAH+TH groups (p<0.05). Likewise, the treatments mitigated the decrease in TAPSE, observed only in the PAH group (p<0.05). Regarding the RV E/A peaks ratio, there was a decrease in the PAH group; only the PAH+HT group showed an improvement in relation to the PAH group (p<0.05) (Table 1 and Figure 1).

**Table 1:** – Morphometric and Echocardiographic Data

Parameters	Control (n=10)	PAH (n=10)	PAH+GJ (n=9)	PAH+TH (n=7)	PAH+TH+GJ (n=10)	Value p
**Morphometric Data**						
Weigth LV (g)	0.64 [0.61-0.65]	0.56 [0.53-0.60]	0.53 [0.52-0.65]	0.62 [0.59-0.66]	0.60 [0.58-0.64]	0.048
Fulton Index (RV/LV+S)	0.27 ± 0.02	0.42 ± 0.07*	0.40 ± 0.07*	0.48 ± 0.10*	0.42 ± 0.07*	≤0.001
**Echocardiographic Data**						
AT/ET	0.26 ± 0.04	0.15 ± 0.05*	0.19 ± 0.07	0.20 ± 0.08	0.15 ± 0.03*	0.002
TAPSE (cm)	0.21 [0.21-0.22]	0.18 [0.16-0.19]*	0.19 [0.18-0.21]	0.20 [0.18-0.21]	0.19 [0.18-0.19]	0.002
E/A	1.12 ± 0.12	0.76 ± 0.06*	0.79 ± 0.15*	1.27 ± 0.46	0.73 ± 0.09*	≤0.000

PAH: pulmonary arterial hypertension; GJ: grape juice; TH: thyroid hormones; RV: right ventricle; LV: left ventricle; AT/ ET: ratio between acceleration time and ejection time of blood flow through pulmonary artery; TAPSE: tricuspid annular plane systolic excursion; E/A: early ventricular filling velocity / late ventricular filling velocity for RV. One-way ANOVA (post Tukey test), data presented as mean ± standard deviation. Kruskal-Wallis’ test (post-Dunn’s) data presented as median and 25^*th*^ and 75^*th*^ percentiles. * significant difference compared to the control group.

**Figure 1:** – Representative image of echocardiographic data in the RV. The TAPSE is marked for the groups (A) CTR: Control, (B) PAH: pulmonary arterial hypertension, (C) PAH+GJ: pulmonary arterial hypertension plus grape juice, (D) PAH+TH: pulmonary arterial hypertension plus thyroid hormones, and (E) PAH+TH+GJ: pulmonary arterial hypertension plus thyroid hormones plus grape juice.

When analyzing the Fulton index, the PAH+TH group showed a significant increase in relation to the PAH+GJ group (p<0.05). No significant difference was observed in the post-test regarding LV weight. However, an increase in the Fulton index was observed in all groups subjected to PAH in relation to the control (p<0.05) (Table 1).

### Oxidative stress markers

Regarding TBARS and ROS levels, there was no significant difference between the groups (Figure 2A and B). The pro-oxidant enzyme xanthine oxidase (XO) showed increased protein expression in the PAH group in relation to the control. Nevertheless, a positive effect of treatments was detected, as treated groups presented a decrease in XO levels in PAH+GJ, PAH+TH and PAH+TH+GJ groups in relation to the PAH group (p<0.05) (Figure 2C). Besides that, the PAH group showed elevated HSP70 expression compared with the control group. However, the PAH+TH and PAH+TH+GJ groups showed a decrease in HSP70 expression in relation to the PAH group (p<0.05) (Figure 2D).

**Figure 2:** – Analysis of oxidative stress markers (A) TBARS, (B) ROS, (C) XO, e (D) HSP70. Data were expressed as mean and standard deviation (n=9-4/group). (*) Significant difference compared to the control group. (#) Significant difference in relation to the PAH group. Statistical analysis of (A) by Welch’s ANOVA with Games Holmes post-test and of (B), (C), (D) and (E) one-way ANOVA with the Tukey post-test (p<0.05). CTR: control; PAH: pulmonary arterial hypertension; PAH+GJ: pulmonary arterial hypertension plus grape juice; PAH+TH: pulmonary arterial hypertension plus thyroid hormones; PAH+TH+GJ: pulmonary arterial hypertension plus thyroid hormones plus grape juice.

### Antioxidant response

When evaluating enzymatic antioxidants, SOD and CAT activity showed no differences between the groups (Figure 3A and B). SOD2 protein expression was decreased in the PAH and PAH+TH+GJ groups in relation to the control (p<0.05). Catalase expression was not different between the groups (Figure 3C and D). Considering the glutathione peroxidase (GPx) activity, PAH animals presented a decrease when compared to the control. However, the activity of this enzyme was increased in the PAH+GJ, PAH+TH and PAH+TH+GJ groups in relation to the control and PAH groups (p<0.05). Moreover, there was a decrease in the levels of sulfhydryl groups in the PAH groups in relation to the control, PAH+GJ, PAH+TH, and PAH+TH+GJ (p<0.05) (Figure 3E and F). Since NRF2 is capable of controlling the transcription of several antioxidant enzymes, the expression of this factor was evaluated. PAH+TH+GJ group showed increased NRF2 protein levels compared with the control group (p<0.05) (Figure 3G).

**Figure 3:** – Antioxidant response: (A) SOD activity, (B) CAT activity, (C) SOD2 protein expression, (D) CAT protein expression, (E) GPX activity, (F) Sulphydryl group, and (G) NRF2 protein expression. Data were expressed as mean and standard deviation (n=10-4/group). (*) Significant difference compared to the control group. (#) Significant difference in relation to the PAH group. Statistical analysis of (B), (C), and (E) by Welch’s ANOVA with Games Holmes post-test and of (A), (C), (D), (F) and (G) one-way ANOVA with Tukey post-test (p< 0.05). CTR: control; PAH: pulmonary arterial hypertension; PAH+GJ: pulmonary arterial hypertension plus grape juice; PAH+TH: pulmonary arterial hypertension plus thyroid hormones; PAH+TH+GJ: pulmonary arterial hypertension plus thyroid hormones plus grape juice.

### Calcium handling proteins

In respect of p-phospholamban protein expression, a decrease was observed in a co-treated group compared to the control group (p<0.05) (Figure 4A). As for total phospholamban levels, there was an increase in the PAH+GJ group in relation to the control and PAH groups. On the other hand, this protein was decreased in PAH+TH and PAH+TH+GJ groups as compared to PAH+GJ group (p<0.05) (Figure 4B). Despite that, when the p-phospholamban/phospholamban ratio was evaluated, there was no significant change between groups (Figure 4C). Concerning SERCA expression, there was an increase in protein levels in the PAH group; however, the PAH+GJ, PAH+TH and PAH+TH+GJ groups showed diminished SERCA protein expression in relation to the PAH group (p<0.05). Regarding ryanodine receptor protein expression, there was a decrease in PAH+TH and PAH+TH+GJ groups in relation to the PAH and PAH+GJ groups (p<0.05) (Figure 4D and E). Concerning the SERCA/total phospholamban ratio, there was no difference between groups (Figure 4F).

**Figure 4:** – Calcium-handling protein. (A) P-phospholamban, (B) Total phospholamban, (C) P-phospholamban/Total phospholamban ratio, (D) SERCA, (E) Ryanodine receptor, and (F) SERCA/Total phospholamban ratio. Data were expressed as mean and standard deviation (n=4/group). (*) Significant difference compared to the control group. (#) Significant difference in relation to the PAH group. ($) Significant difference in relation to the PAH+GJ group. Statistical analysis of (A) by Kruskal-Wallis with Dunn’s post-test, of (B), (D) and (E) by one-way ANOVA with Tukey post-test and of (C) and (F) by Welch’s ANOVA with Games post-test Holmes (p<0.05). CTR: cControl; PAH: pulmonary arterial hypertension; PAH+GJ: pulmonary arterial hypertension plus grape juice; PAH+TH: pulmonary arterial hypertension plus thyroid hormones; PAH+TH+GJ: pulmonary arterial hypertension plus thyroid hormones plus grape juice.

## Discussion

The present work is innovative, considering that, for the first time, it described the association between the protective effect of GJ on pulmonary vessels and the action of TH against RV adverse remodeling in a PAH model. In our experimental protocol, both GJ and TH improved functional parameters such as TAPSE, AT/ET and E/A ratios, as well as modulating the expression of calcium regulatory proteins. Regarding the oxidative stress, xanthine oxidase (XO), 70 kDa heat shock protein (HSP70), and NrF2 were responsive to both GJ and TH administration, which culminated in the restoration of redox homeostasis (Central Illustration).

HAP-induced RV hypertrophy, which corroborates other studies using the same experimental protocol.^22^ Although the RV hypertrophic response is an attempt to overcome the increase in cardiac afterload caused by pulmonary hypertension, its long-term consequences are harmful to cardiac function.^23^ This hypertrophy is associated with changes in AT/ET and E/A ratio, as well as TAPSE. The reduced AT represents a premature closure of the pulmonary valve due to hypertension. The PAH rats also exhibit an extension of ET, leading to a reduced AT/ET ratio in this group. TAPSE analysis, on the other hand, represents a way to assess RV contractility.^24^ PAH showed a reduction in TAPSE, indicating that RV decreased contractile function. Moreover, the E/A ratio showed a significant reduction in the PAH group, indicating a decrease in ventricle compliance and impaired diastolic function. Taken together, these echocardiographic data support that PAH leads to RV failure. The treatment of PAH with GJ and TH showed improvement in functional parameters since in the PAH+HT and PAH+GJ groups, the AT/ET ratio was not different from the control, meaning less resistance in the pulmonary vasculature. Ludke et al.^25^ also demonstrated that treatment for six weeks with GJ prevented PAH-induced AT/ET ratio changes.^25,26^ Regarding the TAPSE, both treatments, isolated or in combination, improved RV contractility. The inotropism improvement can be associated with changes in calcium handling protein expression. Our results showed, especially in the PAH+GJ+TH group, a normalization of calcium handling protein expression, exhibiting values closer to those found in the control group. This suggests that the combination of treatments seems to evidence a cardioprotective potential in PAH. The calcium-handling proteins were also redox-sensitive, indicating the critical role of ROS in the PAH model.

Mosele et al.^27^ and Castro et al.^13^ showed that the protection of the cardiovascular system by GJ and TH treatment, respectively, involved attenuation of oxidative stress. In our study, sulfhydryl levels decreased in PAH rats, indicating a reduction in the non-enzymatic antioxidant capacity of these animals. This context can be detrimental since PAH decreased SOD expression, reducing both non-enzymatic and enzymatic antioxidant protection. On the other hand, although reduced SOD levels were also diminished in the PAH+TH+GJ, TH plus GJ prevented the decrease in sulfhydryl levels and counter-regulated the antioxidant response to the PAH-imposed oxidative stress.

Anion superoxide radicals are converted to hydrogen peroxide, which can play an important role in many signaling pathways.^28^ Nevertheless, supraphysiological hydrogen peroxide levels may overcome antioxidant response and disrupt redox homeostasis.^29^ CAT and GPx are relevant to maintain adequate levels of this ROS. PAH reduced GPx activity, implying an inadequate antioxidant response. A similar profile was found by Dos Santos Lacerda et al.,^30^ in which PAH led to a reduction in GPx levels. Moreover, Sun et al.^31^ showed that patients with systemic sclerosis-associated PAH had reduced GPx. GJ and TH, isolated or combined, were effective in increasing GPx in our study. Corroborating this result, in a gestational experimental model, Proença et al. demonstrated that GJ treatment protected the heart of the fetus against oxidative stress through improvements in GPx.^32^ Bedê et al.^33^also observed antioxidant improvements and increased GPx levels after GJ administration in rats treated with a high-fat diet. These data are associated with TH and GJ treatment-induced increases in NrF2 levels in our study since NrF2 activation results in enhanced expression of antioxidant enzymes and triggers cytoprotective effects.^34^ Therefore, the NrF2 modulation can be suggested as a mechanism of action of GJ and TH in the cardioprotection.

The present study also evaluated XO expression, a protein related to ROS production, which was increased in the PAH group. As a source of ROS, a previous work described a molecular interaction between XO and TLR4 in neutrophil activation, inducing NF-kB translocation and promoting inflammation.^35^ Both GJ and TH treatments, isolated or combined, were effective in reducing XO expression. Corroborating this, Castro et al.^13^ observed a reduction in XO expression in TH-treated infarcted rats; this result was correlated with diminished MyD88 expression, which is a protein related to the inflammatory process.^36^

The disruption in the redox homeostasis triggers a stress response that involves the recruitment of chaperone proteins.^37^ Intracellularly, HSP70 plays a relevant role as a chaperone, promoting protein folding and refolding, as well as exhibiting an anti-inflammatory effect and attenuating cellular stress. In our study, we observed elevated HSP70 immunocontent in the PAH group. The increase in this protein expression can indicate a compensatory mechanism for cardiac damage induced by PAH. GJ and TH administration reduced HSP70 levels, indicating a cellular environment that was less oxidated.

### Limitation of study

Histological evaluation of the RV to verify cardiomyocyte size and collagen deposition would be important in the study of cardiac remodeling induced by PAH. In this sense, we consider the absence of this evaluation in the experimental protocol as a limitation of the study. Furthermore, this report is still in the experimental protocol phase and requires further studies to understand the mechanisms related to the efficacy and safety of this therapeutic approach.

## Conclusion

Our study demonstrates, for the first time, that the GJ with TH treatment, isolated or combined, can improve RV functional and pulmonary vascular parameters of rats with PAH. This cardiovascular protection can be associated with calcium handling protein expression modulation, improving RV inotropism, and promotion of the reestablishment of cellular redox homeostasis. These results are relevant and open a perspective of new therapeutic strategies to mitigate the cardiovascular complications of PAH.
